# Transcriptome and proteome profiling reveals stress-induced expression signatures of imiquimod-treated Tasmanian devil facial tumor disease (DFTD) cells

**DOI:** 10.18632/oncotarget.24634

**Published:** 2018-03-23

**Authors:** Amanda L. Patchett, Richard Wilson, Jac C. Charlesworth, Lynn M. Corcoran, Anthony T. Papenfuss, Bruce A. Lyons, Gregory M. Woods, Cesar Tovar

**Affiliations:** ^1^ Menzies Institute for Medical Research, University of Tasmania, Hobart, Tasmania 7000, Australia; ^2^ Central Science Laboratory, University of Tasmania, Hobart, Tasmania 7001, Australia; ^3^ Walter and Eliza Hall Institute of Medical Research, Parkville, Victoria 3052, Australia; ^4^ Department of Medical Biology, The University of Melbourne, Parkville, Victoria 3010, Australia; ^5^ Division of Cancer Research, Peter MacCallum Cancer Centre, Melbourne, Victoria 3000, Australia; ^6^ Sir Peter MacCallum Department of Oncology, University of Melbourne, Victoria 3000, Australia; ^7^ School of Medicine, University of Tasmania, Hobart, Tasmania 7000, Australia

**Keywords:** imiquimod, Tasmanian devil, devil facial tumor disease, cancer, immunotherapy

## Abstract

As a topical cancer immunotherapy, the toll-like receptor 7 ligand imiquimod activates tumor regression via stimulation of immune cell infiltration and cytotoxic responses. Imiquimod also exerts direct pro-apoptotic effects on tumor cells *in vitro*, but a role for these effects in imiquimod-induced tumor regression remains undefined. We previously demonstrated that cell lines derived from devil facial tumor disease (DFTD), a transmissible cancer threatening the survival of the Tasmanian devil (*Sarcophilus harrisii*), are sensitive to imiquimod-induced apoptosis. In this study, the pro-apoptotic effects of imiquimod in DFTD have been investigated using RNA-sequencing and label-free quantitative proteomics. This analysis revealed that changes to gene and protein expression in imiquimod treated DFTD cells are consistent with the onset of oxidative and endoplasmic reticulum stress responses, and subsequent activation of the unfolded protein response, autophagy, cell cycle arrest and apoptosis. Imiquimod also regulates the expression of oncogenic pathways, providing a direct mechanism by which this drug may increase tumor susceptibility to immune cytotoxicity *in vivo*. Our study has provided the first global analysis of imiquimod-induced effects in any tumor cell line. These findings have highlighted the potential of cell stress pathways as therapeutic targets in DFTD, and will allow for improved mechanistic use of imiquimod as a therapy in both the Tasmanian devil and human cancers.

## INTRODUCTION

Imiquimod (R-837), an imidazoquinoline analogue of guanosine, is a potent immune modifier [1]. Primarily known as an agonist of the viral single stranded RNA sensor toll-like receptor 7 (TLR7), imiquimod has attracted interest in clinical immunotherapy trials for its anti-viral and anti-tumor properties [1–3]. Currently, it is approved by the FDA as a topical immunotherapy against external genital and perianal warts, actinic keratosis and superficial basal cell carcinoma [2]. Successful imiquimod immunotherapy against these and other lesions leads to extensive immune infiltration and subsequent regression via TLR7-dependent cytotoxic mechanisms [4, 5]. TLR7 is expressed at high levels by plasmacytoid dendritic cells (pDCs), which play key roles in the recruitment and activation of cytotoxic immune cells via cytokine and chemokine production [6]. Imiquimod-stimulated pDCs in mouse models can also directly suppress tumor growth and stimulate apoptosis through TLR7-dependent TNF-related apoptosis-inducing ligand (TRAIL) and granzyme B release [7, 8]. Imiquimod may also inhibit immune-regulatory pathways via antagonism of adenosine receptor signalling [9], and activate NLRP3-mediated inflammasome signalling via stimulation of ROS production [10]. This ability of imiquimod to act on the immune response at multiple levels could account for its success as an anti-cancer drug.

It has previously been found that imiquimod has direct effects on tumor cell function *in vitro* and *in vivo*. Studies *in vitro* revealed direct activation of intrinsic apoptosis in cell lines from a range of cancers including malignant melanoma, basal cell carcinoma, squamous cell carcinoma, gastric cancer and endometrial cancer [11–15]. These apoptotic pathways were activated via mechanisms involving oxidative and endoplasmic reticulum (ER) stress, and regulation of the BCL2 family of proteins [14–19]. Autophagic pathways have also been implicated in this process and may contribute to imiquimod-induced death [12, 13, 20]. *In vivo*, tumor growth was directly arrested by topical imiquimod treatment in an immune-compromised mouse model of endometrial cancer [11]. In another study, imiquimod synergized with radiotherapy to enhance tumor regression via mechanisms dependent on the activation of autophagic pathways [21]. Together these results suggest that anti-tumor effects of imiquimod could play roles in tumor regression in cancer patients receiving imiquimod therapy. This is the case for many other anti-cancer drugs, which deregulate oncogenic pathways and alter the expression of silenced and overexpressed genes, allowing for enhanced targeting by the immune system [22]. Further investigation of the molecular changes that occur in imiquimod-treated tumor cells is required to understand pathway regulation in response to this therapeutic molecule.

Devil facial tumor disease (DFTD) refers to two genetically distinct transmissible cancers (DFT1 and DFT2) threatening the survival of the world’s largest carnivorous marsupial, the Tasmanian devil (*Sarcophilus harrisii*). DFTD cells are transferred between devils in a contact dependent manner, where they successfully evade both allogeneic and anti-cancer immune defences to grow and survive in new hosts [23, 24]. Although the development of a prophylactic vaccine against DFTD is being investigated [25, 26], there are currently no prophylactic or therapeutic options for the protection of wild Tasmanian devils from the disease. Our previous studies have found that cell lines established from DFTD tumors are sensitive to cell death induced by imiquimod [27]. Furthermore, TLR7 signalling is functional in the immune system of the Tasmanian devil, suggesting that imiquimod could be an effective DFTD immunotherapy [28, 29]. Thorough investigation of imiquimod treated DFTD cells is required to identify direct imiquimod-induced effects of potential detriment or benefit to tumour clearance in the Tasmanian devil. As these analyses are challenged by a lack of Tasmanian devil specific reagents, the effects of imiquimod in DFTD have been explored in this study at the whole mRNA and protein level using RNA sequencing (RNA-seq) and proteomic mass spectrometry (MS). The DFT1 cell line C5065 was selected as a model line for this analysis, as previous investigations have demonstrated that the response of these cells to imiquimod treatment is representative of other DFTD cell lines [27]. This study will improve the current understanding of DFTD tumorigenesis and survival, and could reveal new targets for DFTD therapy. As a naturally occurring tumor with established mechanisms of immune evasion and survival, DFTD also provides an ideal model for studying the mechanisms of imiquimod action for human application.

## RESULTS AND DISCUSSION

### Molecular changes to imiquimod-treated DFT1 cells

**Figure 1:**
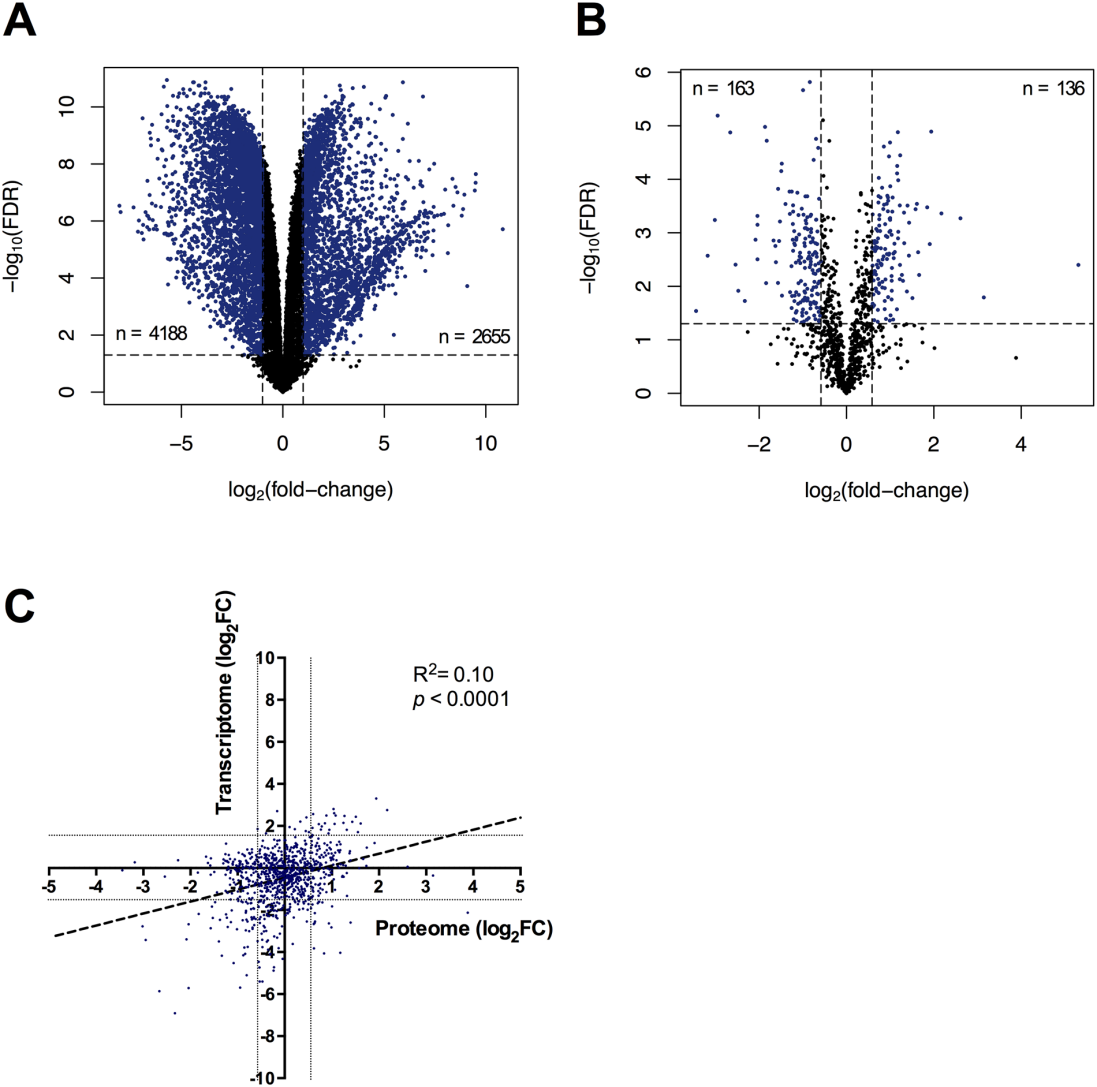
Molecular changes to the transcriptome and proteome of imiquimod treated DFT1 cells C5065 DFT1 cells were treated with imiquimod at 60 μg/ml for 24 or 48 h and analysed by RNA-seq and proteomic MS, respectively. **(A)** Differential mRNA levels and **(B)** differential protein levels between untreated and treated cells were measured and plotted against FDR. Genes with an expression fold-change of greater than ±2 (FDR < 0.05) and proteins with an expression fold-change of greater than ±1.5 (FDR < 0.05) are coloured blue. **(C)** Genes detected at both the mRNA and protein level were compared by log_2_(fold-change) and a linear regression was performed (dotted line). Grey lines represent an mRNA fold-change of ±2 and a protein fold-change of ±1.5.

Imiquimod modulates tumor cell function by mechanisms that are thought to be independent of TLR7 signalling [17], but remain poorly understood in mammalian species including the Tasmanian devil. To understand the molecular pathways regulated by imiquimod in DFT1, we analysed the transcriptome and proteome of untreated and imiquimod-treated cells using RNA-seq and nanoHPLC-MS, respectively. As we previously determined that the percentage of early apoptotic DFT1 cells increases after 48 h of imiquimod treatment, this time point was chosen for promeomics analysis [27]. The transcriptome of imiquimod-treated cells was generated at an earlier time point of 24 h to minimise the extent of RNA degradation for sequencing analysis. After application of quality control filters described in the methods, 13,559 gene transcripts were detected by RNA-seq and 1057 proteins were detected by nanoHPLC-MS in DFT1 cells (Figure 1A and 1B). The molecular landscape of imiquimod-treated and untreated DFTD cells was vastly different, with 2655 (19.58 %) mRNA transcripts significantly up regulated and 4188 (30.89 %) mRNA transcripts significantly down regulated when low stringency cut-offs were used (FDR < 0.05, FC > ±2.0). A more stringent FDR cut-off of 10^−7^ was chosen for down-stream application, reducing the number of differentially expressed genes for functional analysis to 2786 (20.55 %). Statistical analysis of the label-free quantitative proteomics data identified 136 (12.87 %) significantly up regulated proteins and 163 (15.42 %) significantly down regulated proteins after 48 h of imiquimod treatment (FDR < 0.05, FC > ±1.5). Comparison of the measured fold-changes of genes that were detected at both the mRNA and proteome level demonstrated a low but significant correlation of expression trends between the two datasets (R^2^ = 0.10, *p* < 0.0001) (Figure 1C). This low correlation may be a consequence of the different sampling time points for the mRNA and protein analysis, although different mechanisms for regulating mRNA and protein levels can also contribute to low overall correlation in large datasets [30, 31].

To identify functions associated with differentially expressed genes in imiquimod-treated DFT1 cells, gene ontology (GO) analysis was performed. The most significant GO_BP (biological process) terms associated with up regulated genes revealed deregulation of protein folding and activation of the unfolded protein response (UPR) in the endoplasmic reticulum (ER) in response to imiquimod treatment (Table 1A). Other functions associated with ER stress such as apoptosis, autophagy and cholesterol biosynthesis were also positively regulated. Terms associated with genes down regulated by imiquimod indicated that DNA replication and cell cycle were arrested (Table 1B). Many down regulated terms were also associated with the Schwann cell origin of DFT1 cells, suggesting attenuation of normal DFT1 function. Analysis of differentially expressed proteins using DAVID [32, 33] revealed up regulation of protein folding and down regulation of proteins associated with translation, confirming involvement of protein biosynthesis in the ER in response to imiquimod (Table 2A-2B). Proteins associated with the mitochondria and spliceosomes were also positively regulated, and a role for disruption of redox homeostasis in the response to imiquimod was revealed. Together these findings suggest that functional changes that occur in imiquimod-treated DFT1 cells are related to the onset of stress responses and manifest at both the transcriptional and translational level. The principal functions regulated by imiquimod in DFT1 cells are described in detail below.

**Table 1:** Most significant biological process GO terms associated with genes regulated greater than 2-fold in imiquimod-treated DFT1 cells

GO ID	GO term	Term size	Regulated	p-value
A. Up regulated genes				
GO:0035966	response to topologically incorrect protein	181	36	3.00 × 10^−15^
GO:0006986	response to unfolded protein	166	33	4.41 × 10^−14^
GO:0070059	intrinsic apoptotic signaling pathway in response to endoplasmic reticulum stress	56	16	5.64 × 10^−10^
GO:0006984	ER-nucleus signaling pathway	43	13	1.10 × 10^−08^
GO:0035967	cellular response to topologically incorrect protein	141	23	1.73 × 10^−08^
GO:0030968	endoplasmic reticulum unfolded protein response	125	21	4.25 × 10^−08^
GO:0034620	cellular response to unfolded protein	128	21	6.50 × 10^−08^
GO:1902235	regulation of endoplasmic reticulum stress-induced intrinsic apoptotic signaling pathway	29	10	1.37 × 10^−07^
GO:0042594	response to starvation	150	22	2.46 × 10^−07^
GO:0000407	pre-autophagosomal structure	31	10	2.81 × 10^−07^
GO:0050821	protein stabilization	128	20	3.03 × 10^−07^
GO:0009267	cellular response to starvation	113	18	8.79 × 10^−07^
GO:1990440	positive regulation of transcription from RNA polymerase II promoter in response to endoplasmic reticulum stress	11	6	1.91 × 10^−06^
GO:0036003	positive regulation of transcription from RNA polymerase II promoter in response to stress	23	8	2.35 × 10^−06^
GO:0016126	sterol biosynthetic process	56	12	2.43 × 10^−06^
GO:0016241	regulation of macroautophagy	123	18	3.10 × 10^−06^
GO:0006695	cholesterol biosynthetic process	50	11	4.86 × 10^−06^
GO:1902653	secondary alcohol biosynthetic process	51	11	5.97 × 10^−06^
GO:0046685	response to arsenic-containing substance	26	8	6.70 × 10^−06^
GO:0043618	regulation of transcription from RNA polymerase II promoter in response to stress	62	12	7.48 × 10^−06^
**GO ID**	**GO term**	**Term size**	**Regulated**	**p-value**
**B. Down regulated genes**				
GO:0006261	DNA-dependent DNA replication	135	45	4.53 × 10^−16^
GO:0007062	sister chromatid cohesion	124	38	1.70 × 10^−12^
GO:0006270	DNA replication initiation	39	20	6.21 × 10^−12^
GO:0010001	glial cell differentiation	175	44	5.34 × 10^−11^
GO:0007422	peripheral nervous system development	69	24	1.19 × 10^−09^
GO:0061647	histone H3-K9 modification	38	16	3.01 × 10^−08^
GO:0000070	mitotic sister chromatid segregation	132	32	5.59 × 10^−08^
GO:0034329	cell junction assembly	192	40	1.19 × 10^−07^
GO:0007266	Rho protein signal transduction	151	34	1.50 × 10^−07^
GO:0031570	DNA integrity checkpoint	159	35	1.79 × 10^−07^
GO:0032392	DNA geometric change	81	23	1.94 × 10^−07^
GO:0032508	DNA duplex unwinding	76	22	2.46 × 10^−07^
GO:0007160	cell-matrix adhesion	193	39	3.94 × 10^−07^
GO:0042552	myelination	103	26	4.14 × 10^−07^
GO:0007272	ensheathment of neurons	106	26	7.54 × 10^−07^
GO:0008366	axon ensheathment	106	26	7.54 × 10^−07^
GO:0007088	regulation of mitotic nuclear division	133	30	7.64 × 10^−07^
GO:0006271	DNA strand elongation involved in DNA replication	23	11	9.49 × 10^−07^
GO:1901988	negative regulation of cell cycle phase transition	156	33	1.03 × 10^−06^
GO:0090329	regulation of DNA-dependent DNA replication	42	15	1.04 × 10^−06^

**Table 2:** Representative enriched ontological terms associated with proteins regulated greater than 1.5-fold in imiquimod-treated DFT1 cells

Category	Term	No. proteins	Enrichment Score	*p*-Value	FDR
(A) Up-regulated proteins					
GOTERM_MF_DIRECT	poly(A) RNA binding	60	18.53	3.69E-31	5.08E-28
GOTERM_BP_DIRECT	mRNA spicing, via spliceosome	29	16.71	7.28E-25	1.13E-21
GOTERM_CC_DIRECT	nucleoplasm	58	12.71	8.30E-12	1.09E-08
GOTERM_CC_DIRECT	spliceosomal complex	10	9.54	7.99E-08	1.05E-04
GOTERM_BP_DIRECT	protein folding	19	9.18	4.72E-06	2.95E-11
GOTERM_CC_DIRECT	intracellular ribonucleoprotein complex	15	6.26	6.37E-12	8.36E-09
UP_KEYWORDS	mitochondrion	29	6.18	8.83E-09	1.14E-05
GOTERM_CC_DIRECT	endoplasmic reticulum lumen	17	4.74	4.90E-12	6.43E-09
GOTERM_BP_DIRECT	cell redox homeostasis	8	4.35	4.49E-06	6.98E-03
GOTERM_BP_DIRECT	negative regulation of mRNA splicing, via spliceosome	6	3.71	8.16E-07	1.27E-03
**(B) Down-regulated proteins**					
GOTERM_BP_DIRECT	translational initiation	51	45.75	1.62E-67	2.58E-64
GOTERM_CC_DIRECT	cell-cell adherens junction	30	18.55	4.01E-21	5.31E-18
GOTERM_BP_DIRECT	tRNA aminoacylation for protein translation	7	3.91	2.15E-06	3.43E-03
INTERPRO	actin/actin-like conserved site	5	3.81	3.13E-05	4.51E-02
INTERPRO	armadillo-type fold	16	3.57	5.43E-07	7.83E-04
GOTERM_BP_DIRECT	regulation of translational initiation	7	3.34	1.13E-06	1.79E-03
GOTERM_MF_DIRECT	translation initiation factor activity	7	3.23	2.83E-05	3.81E-02
UP_KEYWORDS	Neurodegeneration	11	3.08	1.32E-04	1.68E-01
GOTERM_BP_DIRECT	vesicle mediated transport	8	2.27	6.85E-04	1.09E+0
UP_KEYWORDS	ATP binding	24	2.63	7.46E-04	9.47E-01

### ER stress responses

**Figure 2:**
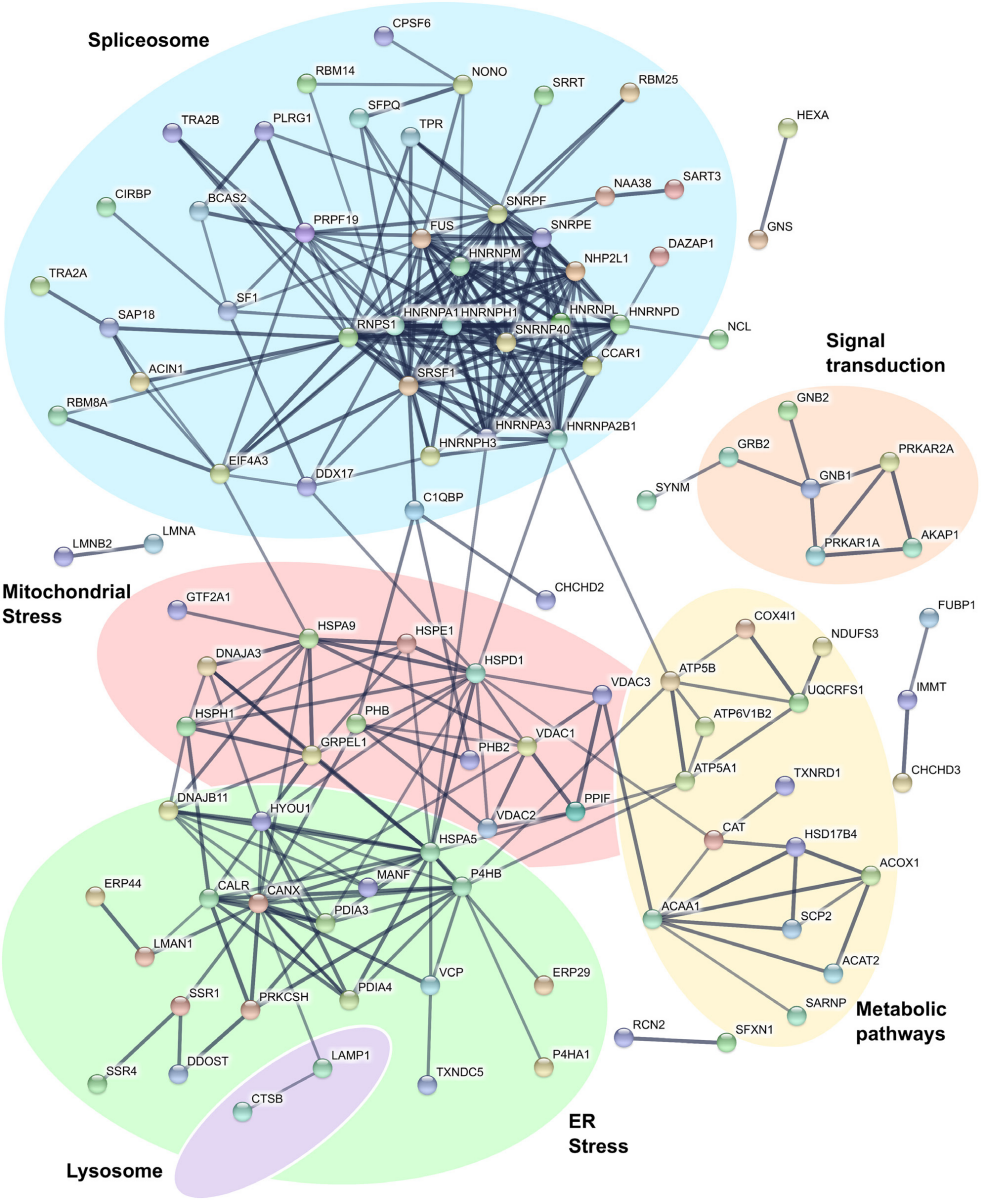
Interactions of proteins up regulated by imiquimod in DFT1 cells C5065 DFT1 cells were treated with imiquimod at 60 μg/ml for 48 h. The proteome of treated and untreated cells was analysed by proteomic MS. Proteins significantly up regulated greater than 1.5-fold (FDR < 0.05) were analysed for protein-protein interactions using the STRING database. Only interactions predicted with high confidence were included in the analyses, and proteins with no predicted interactions were removed. Functional groups were assigned based on scientific literature.

Investigations in tumor cell lines have determined that imiquimod is a strong inducer of oxidative and ER stress responses [16, 17, 19]. GO analysis of the DFT1 transcriptome and proteome confirmed that these responses were also activated in imiquimod-treated DFT1 cells. To further understand these responses, we examined proteins up regulated by imiquimod for protein-protein interactions using the STRING database [34] (Figure 2). In support of previous findings, a network of ER proteins was up regulated in response to imiquimod treatment (green network). These proteins play key roles in ER stress-related functions such as protein re-folding and degradation, protein transport and calcium homeostasis. Binding immunoglobulin protein (BiP; HSPA5; FC = 2.7, *p* = 6.48×10^−4^), a master regulator of ER stress responses in other species [35], was included in this protein network.

**Figure 3:**
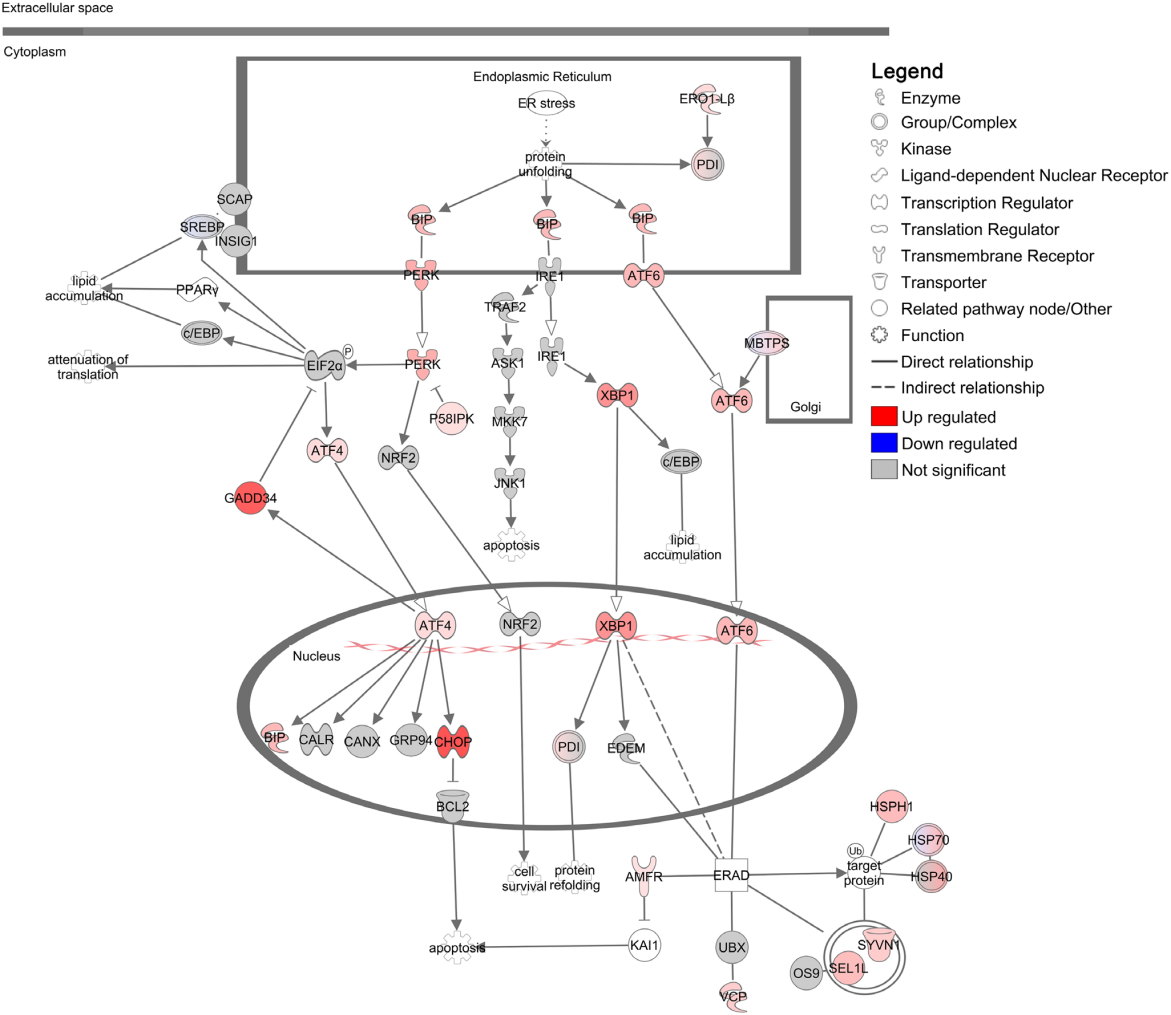
Regulation of the unfolded protein response in DFT1 cells by imiquimod C5065 DFT1 cells were treated with imiquimod at 60 μg/ml for 24 h. The transcriptome of treated and untreated cells was analysed by RNA-seq and IPA was used to predict canonical pathways regulated by imiquimod treatment. The significantly regulated canonical pathway ‘Unfolded protein response’ is shown. Up-regulated genes are coloured red and down-regulated genes are coloured blue.

BiP regulates the UPR, an adaptive response of three key signalling networks, to restore ER homeostasis and promote cell survival during cellular stress (the IRE1-XBP1, ATF6 and PERK-EIF2α-ATF4 pathways). These pathways reduce protein damage and overload within the ER through increased capacity for protein folding (IRE1-XBP1 and ATF6 pathways), removal of terminally misfolded proteins via ER-associated degradation (ERAD) (IRE1-XBP1 and ATF6 pathways) and attenuation of protein translation to mitigate ER protein overload (PERK-EIF2α-ATF4 pathway) [36–38]. To determine whether UPR pathways were activated by imiquimod, we analysed differentially expressed genes that were detected by RNA-seq analysis in more detail using Ingenuity Pathway Analysis (IPA). Analysis of predicted canonical pathways revealed that ‘Unfolded protein response’ (*p* = 4.x10^−09^) and ‘Endoplasmic reticulum stress pathway’ (*p* = 1×10^−06^) were two of the most over-represented and significantly modulated pathways associated with imiquimod treatment in DFT1 cells (Supplementary Table 3). Further examination of the pathway ‘Unfolded protein response’ demonstrated that components of all three UPR pathways were positively regulated at the gene level, suggesting that imiquimod induces significant ER stress in DFT1 cells (Figure 3). This finding was confirmed through quantitative RT-PCR gene expression analysis of the key UPR markers *HSPA5* (CHOP), *XBP1* and *DDIT3* (CHOP) across four imiquimod treated DFT1 cell lines (C5065, 1426, 4906 and half-pea; Supplementary Figure 1). Significant increases in the expression of UPR markers were measured across 72 h of imiquimod treatment in all four lines, confirming that UPR pathways are stimulated by imiquimod in DFT1 cells.

The UPR plays a key role in determination of cell fate during ER stress, and can activate expression of pro-apoptotic CHOP (*DDIT3*) and JNK1 (*MAPK8*) when cell stress and damage cannot be resolved. Specifically, the PERK-ATF4 axis, which regulates pro-apoptotic CHOP (*DDIT3;* FC = 42.6, FDR = 1.91×10^−10^) and pro-survival GADD34 (*PPP1R15A;* FC = 34.0, FDR = 4.13×10^−11^), was positively regulated at the mRNA level in our study and provides the first evidence for ER-stress mediated cell death in imiquimod treated DFT1 cells. Our findings are consistent with activation of both CHOP and GADD34 gene expression, suggesting that ER stress pathways play roles in determining cell fate during imiquimod treatment.

### Mitochondrial stress responses

The UPR is also known to modulate protein expression within the mitochondria, where protein damage can have deleterious effects on metabolic processes [39]. Consistent with a role of the mitochondria in imiquimod-induced cell stress, STRING analysis of proteins up-regulated by imiquimod revealed a network containing mitochondrial VDAC channels (VDAC1, FC = 2.5, *p* = 1.56×10^−3^; VDAC2, FC = 2.1, *p* = 1.76×10^−2^; VDAC3, FC = 1.8, *p* = 5.91×10^−2^) and numerous stress inducible mitochondrial chaperone proteins (HSP10 (HSPE1; FC = 1.7, *p* = 3.74×10^−3^), HSP60 (HSPD1; FC = 2.0, *p* = 3.74×10^−5^), HSP70 (HSPA9; FC = 1.8, *p* = 2.45×10^−5^) and HSP105 (HSPH1; FC = 2.1, *p* = 2.19×10^−4^)) (red network) (Figure 2). Mitochondrial chaperones can be activated by disruptions to oxidative phosphorylation that produce an excess of unassembled respiratory chain components in the mitochondria [40]. Key protein subunits of respiratory chain complexes III (UQCRFS1; FC = 8.8, *p* = 1.61×10^−2^), IV (COX4I1; FC = 1.7, *p* = 8.78×10^−3^) and V (ATP5A1, FC = 1.5, *p* = 9.40×10^−4^; ATP5B, FC = 2.5, *p* = 1.62×10^−3^) were up regulated in DFT1 cells (yellow network), demonstrating involvement of oxidative phosphorylation in the response to imiquimod treatment.

Disruptions to oxidative phosphorylation could explain a shift in redox balance in imiquimod treated DFT1 cells. Proteins associated with cellular responses to oxidative stress such as catalase (CAT; FC = 2.2, *p* = 7.76×10^−5^) and thioredoxin reductase 1 (TXNRD1; FC = 2.1, *p* = 4.02×10^−4^) were up regulated, suggesting that elevated levels of reactive oxygen species (ROS) are present after imiquimod treatment (Figure 2). ROS could shuttle between the ER and mitochondria to alter protein homeostasis and further exacerbate the ER stress response [41]. Calcium released from the ER during stress is also taken up by mitochondrial ion channels such as VDACs, and can further stimulate ROS production [42]. Such a mechanism of action is supported by previous observations of heightened levels of intracellular ROS and depletion of ER calcium in imiquimod-treated tumor cells [17, 43]. The involvement of ROS in the stress response to imiquimod likely explains the susceptibility of tumor cells to imiquimod-induced apoptosis. ROS accumulates at a faster rate in highly metabolic tumor cells, leading to detrimental conditions that promote apoptotic pathways [44]. Pro-survival mechanisms such as the UPR are also constitutively active in tumors to cope with high metabolic demands, and additional production of ROS is more likely to overwhelm these mechanisms to promote a pro-apoptotic state [45].

### Tumorigenesis

**Figure 4:**
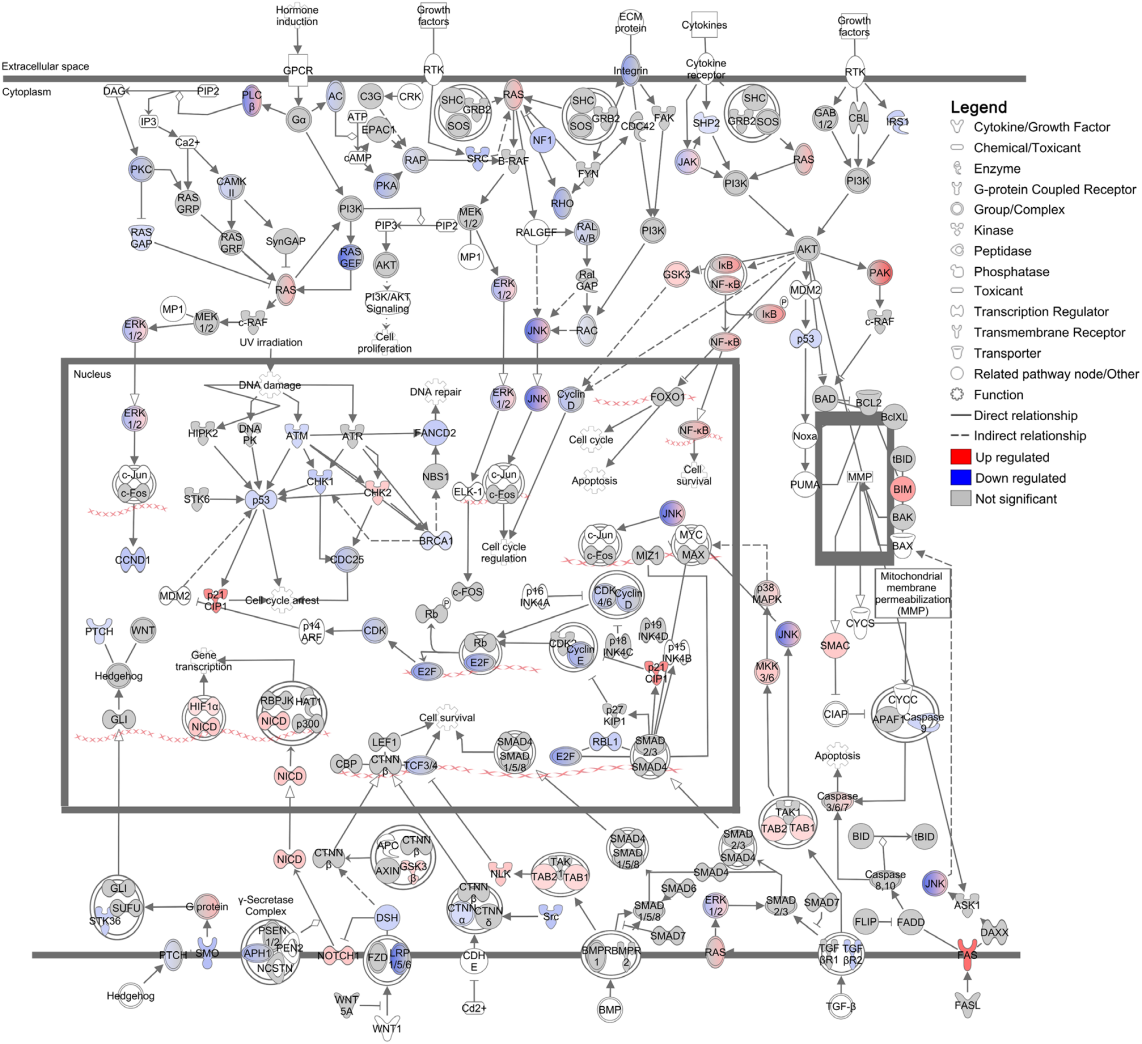
Regulation of ‘Molecular mechanisms of cancer’ canonical pathway in DFT1 cells by imiquimod C5065 DFT1 cells were treated with imiquimod at 60 μg/ml for 24 h. The transcriptome of treated and untreated cells was analysed by RNA-seq and IPA was used to predict canonical pathways regulated by imiquimod treatment. The canonical pathway most significantly associated with genes regulated by imiquimod treatment is shown. Up-regulated genes are coloured red and down-regulated genes are coloured blue.

The UPR modulates translation to prevent protein overload within the ER and to promote pro-survival responses during cellular stress [38, 46]. This attenuation of translation is controlled by PERK (EIF2AK3), which phosphorylates the translational regulator EIF2A leading to preferential biosynthesis of stress-related proteins and suppression of overall protein translation [38, 46]. To determine how these translational changes alter the DFT1 landscape, we further analysed canonical pathways that were predicted by IPA to be regulated by imiquimod treatment. Investigation of these pathways revealed an overall suppression of tumorigenesis in imiquimod-treated DFT1 cells, with down regulation of numerous pathways involved in tumorigenic processes such as proliferation, resistance to apoptosis, angiogenesis, immune evasion, migration and invasion (Supplementary Table 3) [47–50]. Examples of these included ‘Integrin Signalling’ (p = 3×10^−10^), ‘STAT3 Pathway’ (p = 1×10^−08^) and ‘Protein Kinase A Signalling’ (p = 2×10^−08^). The canonical pathway most significantly associated with the RNA-seq dataset was ‘Molecular Mechanisms of Cancer’ (p = 6×10^−13^) (Figure 4). Analysis of this pathway in detail revealed additional networks by which imiquimod influenced tumor growth, with deregulation of cyclin signalling and strong up-regulation of p21^CIP1^ (*CDKN1A;* FC = 12.1, FDR = 2.69×10^−10^), an inducer of replicative senescence, at the mRNA level. Genes encoding pathways that promote cell cycle, DNA repair and proliferation were also negatively regulated, including Ras, p53, BRCA1, integrin, phospholipase C and Hedgehog signalling pathways. Other pathways, such as NFκB, p38 MAPK and NOTCH1 signalling pathways were positively regulated at the mRNA level, and may play roles in the response to imiquimod treatment.

With such potent effects on tumorigenesis, imiquimod may increase the ability of immune cells to mount anti-tumor cytotoxic responses *in vivo*. In support of this, the death receptor *FAS* was present at only low mRNA levels in the transcriptome of untreated DFT1 cells and was up regulated by imiquimod treatment (FC = 23.7, FDR = 1.12×10^−9^; Figure 4). *FAS* expression is frequently suppressed in tumor cells to evade immune-mediated cytotoxicity and is functionally up regulated in an immune-independent manner in response to other anti-cancer drugs such as cisplatin and doxorubicin [51]. Up regulation of *FAS* expression in imiquimod-treated DFT1 cells could increase their susceptibility to immune-mediated cytotoxicity. In addition, the anti-phagocytic gene *CD200* (FC = 0.1, FDR = 1.08×10^−9^), the angiogenic gene *VEGFA* (FC = 0.5, FDR = 8.24×10^−7^) and the checkpoint inhibitor *CD276* (FC = 0.2, FDR = 2.20×10^−8^) were down regulated at the mRNA level in DFT1 cells by imiquimod treatment (Supplementary Table 1). As these molecules suppress immune responses, their down regulation provides further evidence for increased immunogenicity of imiquimod-treated DFT1 cells. Other regulatory molecules such as PDL1 (*CD274*) exhibited low or no expression at baseline in DFT1 cells and were not up regulated at the mRNA level by imiquimod.

### Cell cycle arrest

**Figure 5:**
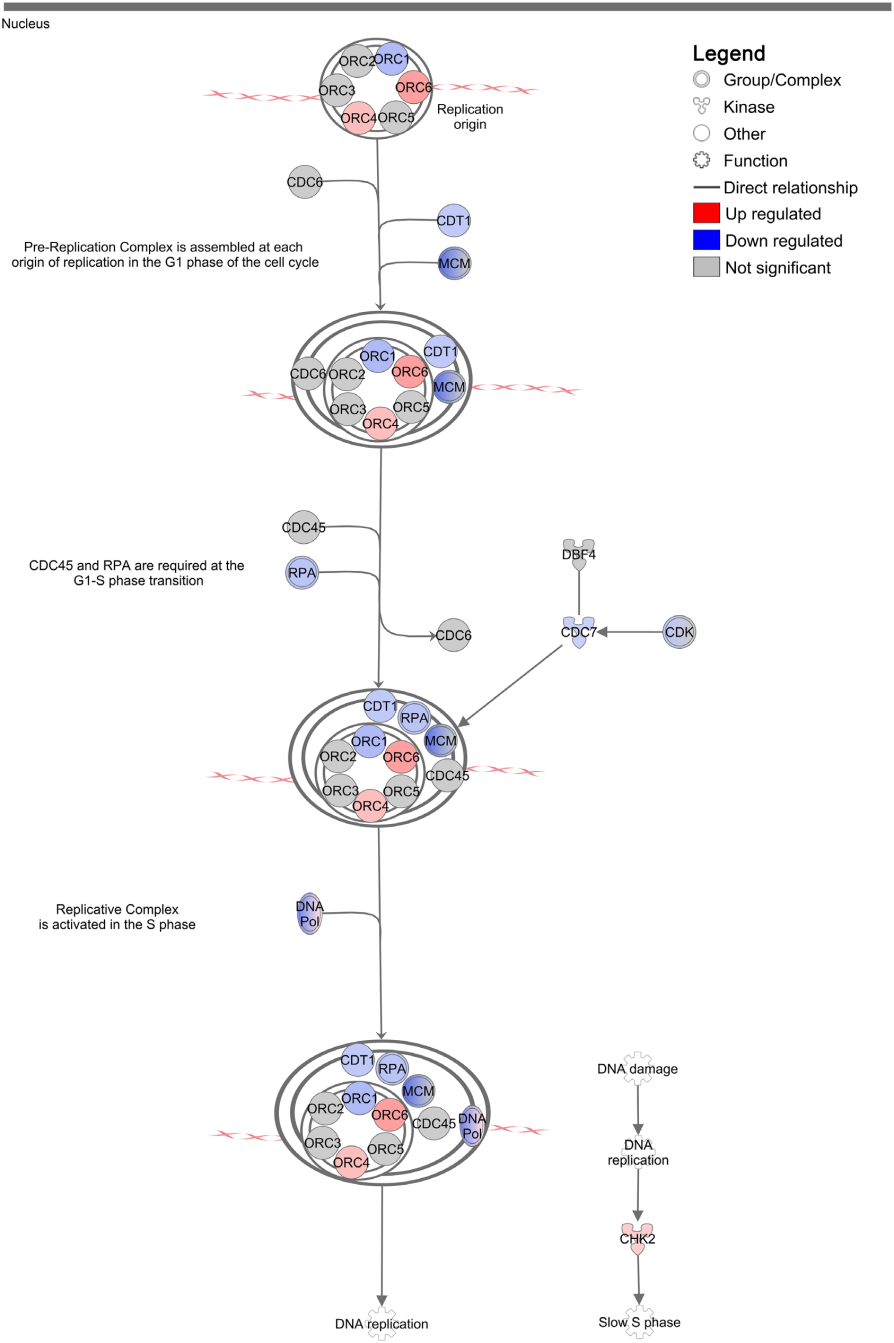
Regulation of ‘Cell cycle control of chromosomal replication’ canonical pathway in DFT1 cells by imiquimod C5065 DFT1 cells were treated with imiquimod at 60 μg/ml for 24 h. The transcriptome of treated and untreated cells was analysed by RNA-seq and IPA was used to predict canonical pathways regulated by imiquimod treatment. The highly regulated pathway ‘Cell cycle control of chromosomal replication’ is shown. Up-regulated genes are coloured red and down-regulated genes are coloured blue.

Previous studies in animal models have demonstrated immune-independent suppression of tumor growth in response to imiquimod treatment *in vivo* [11]. This suppression of tumor growth is likely the result of UPR-induced cell cycle arrest, which allows time for cell damage to be repaired prior to cell division. In support of this, genes found to be positively regulated in DFT1 cells at the mRNA level by imiquimod included the cyclin/cyclin dependent kinase (CDK) inhibitor p21^CIP1^ (*CDKN1A;* FC = 12.1, FDR = 2.69×10^−10^), an inducer of replicative senescence at the G1/S phase (Figure 4). Consistent with these findings, network analysis of the DFT1 transcriptome using IPA revealed that the canonical pathway ‘Cell cycle control of chromosomal replication’ was the second most significant pathway associated with imiquimod treatment (p = 3×10^−11^; Figure 5). Analysis of this pathway revealed strong suppression of mRNA encoding necessary components of chromosomal replication machinery, including MCM (including *MCM6;* FC = 0.2, FDR = 4.07×10^−11^), RPA (*RPA1;* FC = 0.2, FDR = 5.92×10^−10^), CDT1 (*CDT1;* FC = 0.2, FDR = 1.53×10^−9^) and DNA polymerase (including *POLA1;* FC = 0.2, FDR = 3.44×10^−10^). CDKs, which are required for cell cycle progression, were also highly suppressed at the mRNA level. CHK2 (*CHEK2*), an upstream negative regulator of CDK activity at the G1/S phase, was up regulated (FC = 2.9, FDR = 3.43×10^−8^). In comparison, genes encoding the G2/M cyclin/CDK inhibitor CHK1 (*CHEK1*) were down regulated at the mRNA level after 24 h of imiquimod treatment (FC = 0.3, FDR = 3.72×10^−9^). Together these results indicate that imiquimod suppresses DFT1 cell cycle at the G1/S phase after 24 h of treatment. This finding supports previous analysis of imiquimod action in DFT1 cell lines, which demonstrated significant inhibition of cell proliferation after treatment [27].

G1/S cell cycle arrest during ER stress is controlled through PERK-dependent preferential translation of p53 (*TP53*) [52]. Unexpectedly, p53 was down regulated in imiquimod-treated DFT1 cells at the mRNA level (FC = 0.3, FDR = 3.77×10^−9^; Supplementary Table 1), opposing its role in imiquimod-induced G1/S arrest. In studies by Huang and colleagues, expression of p53 was up regulated by imiquimod in tumor cells at 24 h and was required for more rapid onset of apoptosis when compared to p53 knockdown cells [18]. Although p53 was down regulated in DFT1 cells by imiquimod treatment, targets of p53 such as *MDM2*, *PTEN* and *FAS* were up regulated in the transcriptome, suggesting that p53 function was not lost. As such, the apparent down regulation of p53 in imiquimod-treated DFT1 cells could be time-dependent, and may represent a negative feedback mechanism to promote cell survival during the early stages of treatment. Alternatively, p53-independent activation of p21^CIP1^ (*CDKN1A*), via mechanisms involving CHK2 (*CHEK2*), has been reported in other models and could play a role in G1/S cell cycle arrest in DFT1 cells [53].

### Autophagy

**Figure 6:**
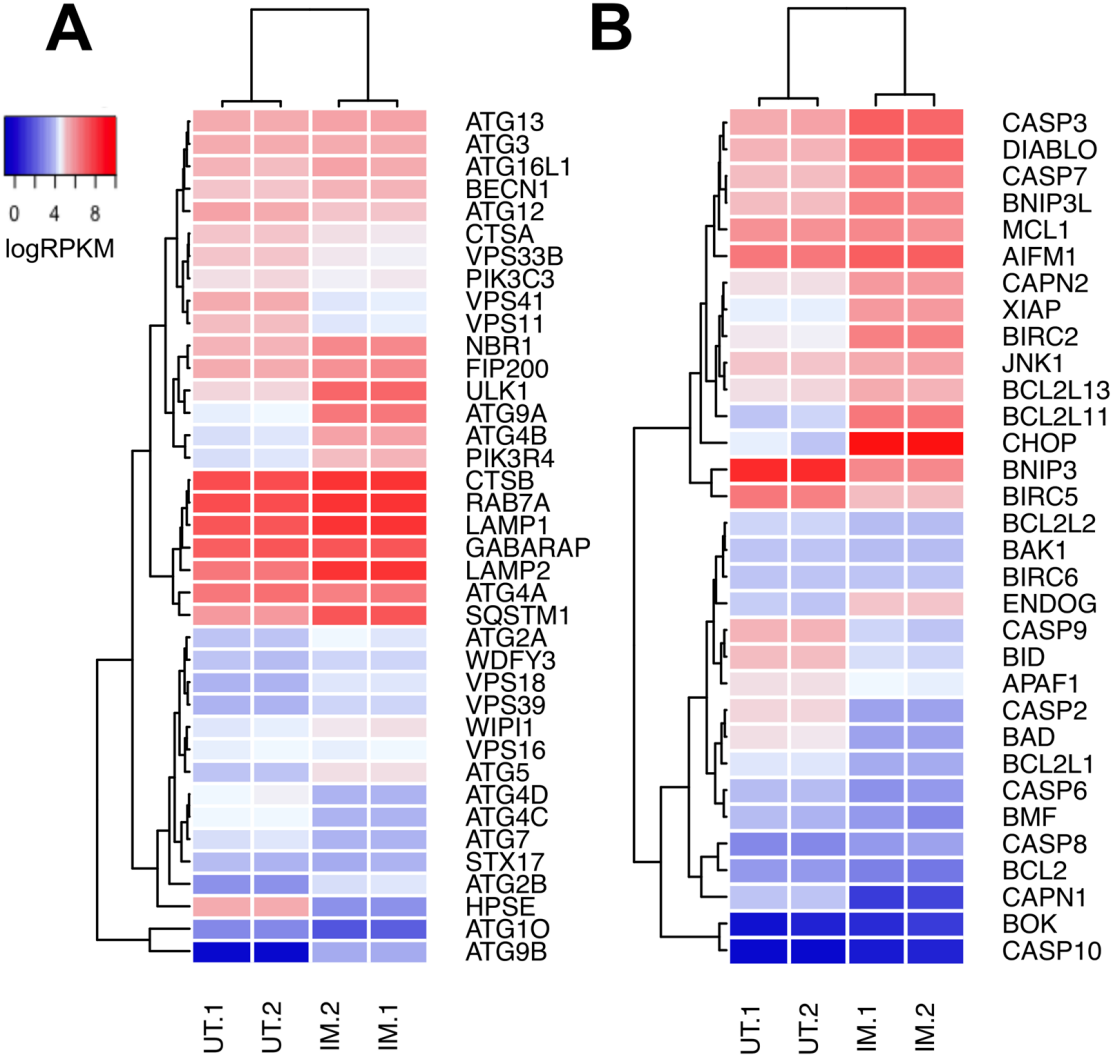
Relative expression of genes associated with autophagy and apoptosis in DFT1 cells after imiquimod treatment C5065 DFT1 cells were treated with imiquimod at 60 μg/ml for 24 h. The transcriptome of treated and untreated cells was analysed by RNA-seq. Relative expression of genes associated with **(A)** autophagic pathways and **(B)** apoptotic pathways in untreated (UT) and imiquimod-treated (IM) cells was visualized on heat maps generated by Euclidean clustering. High gene expression is represented in red and low gene expression is represented in blue.

It is well established that tumor cells treated *in vitro* with imiquimod undergo cell death [14, 16]. In addition to apoptosis, autophagy has been implicated in cell death pathways [12, 16, 19]. In support of a role for autophagy in the response of DFT1 cells to imiquimod treatment, our proteomic analysis revealed up regulation of the lysosomal proteins CTSB (FC = 1.8, *p* = 1.05×10^−2^) and LAMP1 (FC = 1.6, *p* = 1.50×10^−3^) (Figure 2). To further investigate the involvement of autophagy in these treated cells, mRNA expression of genes with known roles in the regulation and execution of autophagy was analysed by hierarchical clustering and represented using a heat map. Many autophagic genes, including class III PI3K (*PIK3C3*), an activator of autophagy, were expressed in both untreated and imiquimod-treated DFT1 cells at the mRNA level (Figure 6A). Furthermore, mRNA encoding P150 (*PIK3R4*), which is required for stabilisation and correct function of the class III PI3K complex, was positively regulated in response to imiquimod treatment (FC = 2.3, FDR = 5.67×10^−9^). Other critical genes required for formation of the autophagosome (*BECN1*, *ATG9A* and *ATG2A*), autophagosome and lysosome fusion (*RAB7A*) and lysosomal protein degradation (*LAMP1, LAMP2* and *CTSA*) were either highly expressed in both untreated and treated cells, or were up regulated in response to imiquimod treatment, suggesting that autophagic pathways were active.

During ER stress, autophagic gene expression is stimulated by PERK-ATF4-mediated transcriptional activity [54]. Autophagy is then activated via the IRE1-JNK axis, which mediates phosphorylation of anti-apoptotic BCL2 and induces its dissociation from the autophagy inducer beclin-1 [55, 56]. Although autophagic pathways have the potential to induce cell death, they are initially activated as a protective anti-apoptotic mechanism by which damaged proteins and organelles are degraded and recycled to minimize cellular stress [55]. In support of this, genes encoding key inhibitors of apoptosis including *MCL1*, XIAP (*ENSSHAG00000016328*) and BIRC2 (*ENSSHAG00000011782*) were highly expressed at the mRNA level in tandem with autophagic genes after 24 h of imiquimod treatment (Figure 6B). These results suggest that autophagic pathways may play a role in promoting and prolonging DFT1 survival after imiquimod treatment.

### Apoptosis

Unresolved cellular stress and prolonged activation of the UPR leads to stimulation of apoptotic pathways. This can occur via direct UPR stimulation of CHOP (PERK-EIF2a-ATF4 pathway) and JNK1 (IRE1-ASK1 pathway), which regulate pro and anti-apoptotic proteins such as BIM, BCL2 and XIAP to stimulate apoptosis [57–59]. An additional mechanism of stress-mediated apoptosis is activated through the calcium-dependent pro-apoptotic protein calpain after ER calcium depletion [60, 61]. Previous analyses have shown that DFT1 cell lines undergo apoptosis after sustained treatment with imiquimod [27]. Direct measurement of pro- and anti-apoptotic factors in this previous study revealed down regulation of anti-apoptotic genes and up regulation of the pro-apoptotic gene BIM (*BCL2L11*) at the mRNA level in response to imiquimod treatment. To further understand these apoptotic pathways, genes associated with apoptosis were also analysed by hierarchical clustering and visualized using a heat map (Figure 6B). As demonstrated in Figure 3, CHOP (*DDIT3;* FC = 42.6, FDR = 1.91×10^−10^) was highly up regulated at the gene level in imiquimod-treated DFT1 cells, while *JNK1* exhibited high constitutive expression that was unchanged after treatment. Isoforms of calpain were differentially regulated, with strong suppression of the μ-isoform *CAPN1* (FC = 0.04, FDR = 2.62×10^−9^) and up regulation of the m-isoform *CAPN2* (FC = 2.5, FDR = 4.74×10^−9^) at the mRNA level. M-calpain can directly activate apoptosis via cleavage of caspase 12, and also interacts with the IRE1-ASK1 pathway during ER stress to promote JNK1-mediated apoptosis [61]. In a previous study, individual inhibition of JNK1, CHOP and calpain pathways slowed but did not prevent imiquimod-induced cell death [16]. This finding suggests that cooperation of these pathways is required for activation of apoptosis after imiquimod treatment.

Mitochondrial pro-apoptotic genes such as *BCL2L11* (FC = 7.7, FDR = 9.56×10^−10^), *DIABLO* (FC = 3.1, FDR = 2.59×10^−8^) and *ENDOG* (FC = 1.8, FDR = 8.85×10^−4^) were also positively regulated at the gene level by imiquimod in DFT1 cells and likely play roles in the activation of apoptotic pathways (Figure 6B). Specifically, *ENDOG* encodes a key inducer of caspase-independent apoptosis and DNA fragmentation during sustained endogenous oxidative stress, and could be stimulated by imiquimod-induced ROS [62, 63]. Activation of apoptotic pathways also requires the spliceosome, which alternatively splices numerous apoptotic regulators to modulate their pro- and anti-apoptotic functions [64]. This regulation is evident after imiquimod treatment, with a large network of proteins involved in mRNA splicing and processing identified by STRING interaction analysis (blue network) (Figure 2). These include all three members of the apoptosis and splicing associated protein (ASAP) complex (ACIN1, FC = 2.5, *p* = 4.20×10^−4^; SAP18, FC = 3.0, *p* = 3.63×10^−4^; RNPS1, FC = 2.3, *p* = 9.58×10^−4^), which is involved in the alternative splicing of regulators of apoptosis including caspases and the members of the BCL2 protein family [64].

### Mechanism of action

Although pathways by which imiquimod modulates cell death have been identified, little is understood as to how these mechanisms are activated. As a potent agonist of TLR7, it is tempting to hypothesize that imiquimod induces ER stress and apoptosis in tumor cells via TLR7 signalling. However, studies have shown that tumor cell lines lacking TLR7 are also sensitive to imiquimod-induced autophagy and apoptosis [17, 19, 43]. We previously reported that DFTD cells also do not express TLR7 at any stage of imiquimod treatment, suggesting that imiquimod acts via a TLR7-independent mechanism in DFT1 cells [27]. Myeloid cells treated with imiquimod undergo a ROS burst that is activated via direct inhibition of NAD(P)H quinone dehydrogenase 2 (NQO2) and complex I of the respiratory chain [10]. Similar direct inhibition of complex I in highly metabolic tumor cells would shift oxidative homeostasis, leading to the activation of mitochondrial and ER stress responses. This is the case for other known inhibitors of complex I, which demonstrate anti-cancer effects involving substantial ROS production and ER stress [65, 66]. Decreased oxidative respiration and ATP depletion have been measured in imiquimod-treated tumor cells, providing evidence for respiratory chain inhibition [19, 43]. In contrast, key subunits of complexes III, IV and V were up regulated at the mRNA level in imiquimod-treated DFT1 cells (Figure 2). These complexes may have been up regulated as a compensatory mechanism for ATP depletion, and could add to oxidative stress at the mitochondria in a vicious stress cycle. Studies targeting specific key elements are required to further investigate the mechanism of imiquimod action in tumor cells.

### Implications for imiquimod therapy

The potent anti-tumor effects of imiquimod in DFT1 cells have highlighted the potential of stress responses as targets for deregulation of DFTD tumor cell survival. Indeed, our results showed that pro-survival pathways including autophagy, the UPR and antioxidant signalling were overwhelmed by imiquimod-induced cell stress in DFT1 cells, demonstrating the potential of stress-inducing drugs as therapeutic agents in DFTD. Imiquimod has also been found to be an effective immune adjuvant in the Tasmanian devil [28, 29], supporting its use as a DFTD immunotherapy. Although imiquimod would be difficult to distribute among a wild devil population, the drug could be available for opportunistic treatment of captured devils affected by the disease. As DFTD tumors are allografts [24], it is possible that only a short period of imiquimod treatment would be required to break immune tolerance and induce allogeneic immunity. Captive trials are required to determine whether imiquimod is an effective immunotherapy in the Tasmanian devil.

Studies of imiquimod action in DFTD also provide valuable insights into the response of human tumor cells to direct imiquimod treatment. While previous studies have elucidated a role for imiquimod in the activation of stress responses in human cancer cell lines [16, 17], our analyses have used DFTD as a tumor model to highlight the key molecules and pathways regulated at a global scale. In human cancer therapy, combinatorial immunotherapies have demonstrated promise for their ability to target multiple pathways and overcome immune resistance [67]. Through an improved understanding of pathways regulated by imiquimod, the approach used in our analysis will allow for mechanistic identification of potential therapeutic targets and drug combinations involving imiquimod treatment. Our analysis also provides evidence that imiquimod exerts suppressive effects against pathways involved in the normal functioning of tumor cells, including those involved in invasion, cell migration and immune evasion. These suppressive effects could improve the ability of the immune system to target tumor cells, perhaps aiding tumor rejection *in vivo*. This study provides a basis for further exploration of imiquimod as a therapy in cancer.

## MATERIALS AND METHODS

### Cell lines and imiquimod treatment

The DFT1 cell lines C5065, 1426, 4906 and half-pea were provided by A.-M. Pearse and K. Swift of the Tasmanian Department of Primary Industries, Parks, Water and Environment (DPIPWE). A total of 1 × 10^6^ cells were plated per well of a 12-well cell culture plate (Corning, New York, USA) in 1 ml of RPMI culture medium (Thermo Fisher Scientific, Waltham, USA)/10% foetal calf serum (FCS). Cells were treated with imiquimod (Sigma-Aldrich, St Louis, USA) prepared as previously described [27], at 60 μg/ml in a 35 °C, 5% CO_2_ humidified incubator. As it was previously determined that DFT1 cells undergo imiquimod-mediated cell death after 24-96 h incubation with the drug [27], cells were treated for 24 h for RNA-seq to ensure mRNA integrity. A longer treatment of 48 h was used for proteomics analysis in order to understand the changes occurring as the cells enter apoptotic pathways. For confirmation of findings by quantitative PCR, cells were treated for 0, 8, 16, 24, 48 or 72 h, and PCR was performed according to previously described methods [27]. Untreated controls were included in all analyses.

### Next generation mRNA sequencing

RNA was extracted from untreated and imiquimod-treated C5065 DFT1 samples using the RNeasy® Mini Kit (QIAGEN Bioinformatics, Hilden, Germany), according to the manufacturer’s instructions. RNA integrity was assessed using a Eukaryotic RNA 6000 Nano Kit and 2100 Bioanalyzer (Agilent, Santa Clara, USA). 100 base-pair single-read mRNA sequencing was performed on the Illumina Hiseq-2000 platform (Illumina, San Diego, USA). The RNA-seq data have been deposited to the NCBI Sequence Read Archive (BioSample accessions SAMN07998608, SAMN07998609, SAMN07998610, SAMN07998611) with links to BioProject accession number PRJNA416378 in the NCBI BioProject database (https://www.ncbi.nlm.nih.gov/bioproject/). Sequence quality was assessed using fastqc (http://www.bioinformatics.babraham.ac.uk/projects/fastqc). Sequencing reads were aligned to the Tasmanian devil reference genome (7.0.82) using subread [68], and counts were summarized into genes using featureCounts [69]. Differential expression analysis of gene counts was performed using R version 3.3.2. For this analysis, technical replicates were combined and genes with less than 20 aligned reads across all samples were removed. Expression levels were normalized by upper quartile normalisation using EDAseq [70, 71]. Differential expression was calculated using limma/voom [72] (Supplementary Table 1). Volcano plots of differential gene expression data were created using the R plot function, and heat maps were produced using heatmap.2/gplots (https://CRAN.R-project.org/package=gplots) using hierarchical clustering by Euclidean distance. Gene ontology (GO) analysis of genes up and down regulated greater than 2-fold was performed using limma/goana [73, 74] (Supplementary Table 2A-2B). A stringent FDR cut-off of 10^−7^ was applied to this analysis to include only the most significantly altered genes. To remove general and repetitive GO terms, those with a gene size of greater than 200 were filtered from the analysis. Differential gene expression data was further analysed using Ingenuity Pathway Analysis (IPA; QIAGEN) [75]. Canonical pathways associated with differentially expressed genes were generated using the FDR cut-off of 10^−7^ (Supplementary Table 3).

### Nano-liquid chromatography and LTQ-Orbitrap tandem mass spectrometry

Triplicate cultures of untreated and imiquimod-treated C5065 DFT1 cells were lysed in 700 μl of protein extraction buffer (7M urea/2M thiourea in 40 mM Tris with protease inhibitor, pH 8.0) for 2 h at 4 °C. Proteins were precipitated from the lysate with 9 x the volume of 100 % ethanol overnight at −20 °C. Proteins were reconstitutes in protein extraction buffer and concentration was determined using the EZQ® Protein Quantification Kit (Thermo Fisher Scientific, Waltham, USA), according to the manufacturer’s instructions. Precipitated proteins were sequentially reduced, alkylated and digested with proteomics-grade trypsin from porcine pancreas (Sigma-Aldrich, St Louis, USA) as previously described [76]. Tryptic peptides equivalent to ~ 1 μg of digested protein were separated using an Ultimate 3000 nano RSLC system (Thermo Fisher Scientific, Waltham, USA). Peptides were first concentrated on a 20 mm x 75 μm PepMap 100 trapping column (3 μm C18) at a flow rate of 5 μl/min, using 98 % water, 2 % acetonitrile and 0.05% TFA. Peptides were then separated on a 250 mm x 75 μm PepMap 100 RSLC column (2μm C18) at a flow rate of 300 nL/min. A 180 minute gradient from 98% mobile phase A (0.1% formic acid in water) to 50% mobile phase B (0.08% formic acid in 80% acetonitrile and 20 % water) comprised the following steps: 2-10% B over 10 min, 10-40% B over 120 min, 40-50% B over 10 min, holding at 95% B for 10 min then re-equilibration in 2% B for 15 min. The nanoHPLC system was coupled to an LTQ-Orbitrap mass spectrometer equipped with nanopray Flex ion source (Thermo Fisher Scientific, Waltham, USA) and controlled using Xcalibur 2.1 software. MS scans were acquired from 460-2000 m/z at a resolution of 30,000 and MS/MS spectra were acquired in data-dependent mode using a Top8 method and 30-second dynamic exclusion of fragmented peptides, as described [77]. The mass spectrometry proteomics data have been deposited to the ProteomeXchange Consortium via the PRIDE [78] partner repository with the dataset identifier PXD007135.

### Protein identification and analysis

Data files were imported into MaxQuant version 1.5.1.2 (http://maxquant.org/) and MS/MS spectra were searched using the Andromeda search engine against the complete *Sarcophilus harrisii* UniProt reference proteome (ID 0000007648; updated on 11/02/2016) comprising 22,388 protein entries. Default settings for protein identification by LTQ-Orbitrap MS/MS and label-free quantitation (LFQ) included a maximum of two missed cleavages, mass error tolerances of 20 ppm then 4.5 ppm for initial and main peptide searches, respectively, 0.5 Da tolerance for fragment ions, variable methionine oxidation and fixed carbamidomethylation. A default false discovery rate (FDR) of 0.01 was used for peptide-spectrum matches and protein identification. The MaxQuant algorithm MaxLFQ was used for peptide intensity determination and normalization [79], using pair-wise comparison of unique and razor peptide intensities and a minimum ratio count of 2. The proteinGroups output file generated by MaxQuant analysis was processed as follows: The normalized label-free quantification (LFQ) intensity values, MS/MS counts and the numbers of razor and unique peptides for each of the identified proteins were imported into Perseus software version 1.5.031 (http://perseus-framework.org/). Protein groups identified either as potential contaminants (prefixed with CON_), identified by modified site only, by reverse database matching or on the basis of a single matching peptide were removed. LFQ intensity values were then log_2_–transformed and then a filter applied to include only proteins detected in a minimum of the three untreated or imiquimod treated replicates. Missing values were replaced with random intensity values for low-abundance proteins based on a normal distribution of protein abundances using default MaxQuant parameters. To determine proteins that were significantly altered in abundance between treatments a two-sided *t*-test with a permutation-based FDR of 0.05 was applied, using 250 randomizations and a minimum fold-change cut-off of 1.5 (Supplementary Table 4). Volcano plots of protein expression data were created using the plot function in R. Gene set testing of significantly up and down regulated proteins was performed using the Functional Annotation Clustering tool of DAVID version 6.8 [32, 33] (Supplementary Table 5A-5B). As there is a paucity of functional information is available for the Tasmanian devil, protein lists were analysed using the *Homo sapiens* species database, with medium stringency. Protein-protein interactions between up regulated proteins were identified using the STRING Database version 10.5 [34]. Only interactions predicted with high confidence (interaction score ≥ 0.700) were included in the analysis.

## CONCLUSIONS

**Figure 7:**
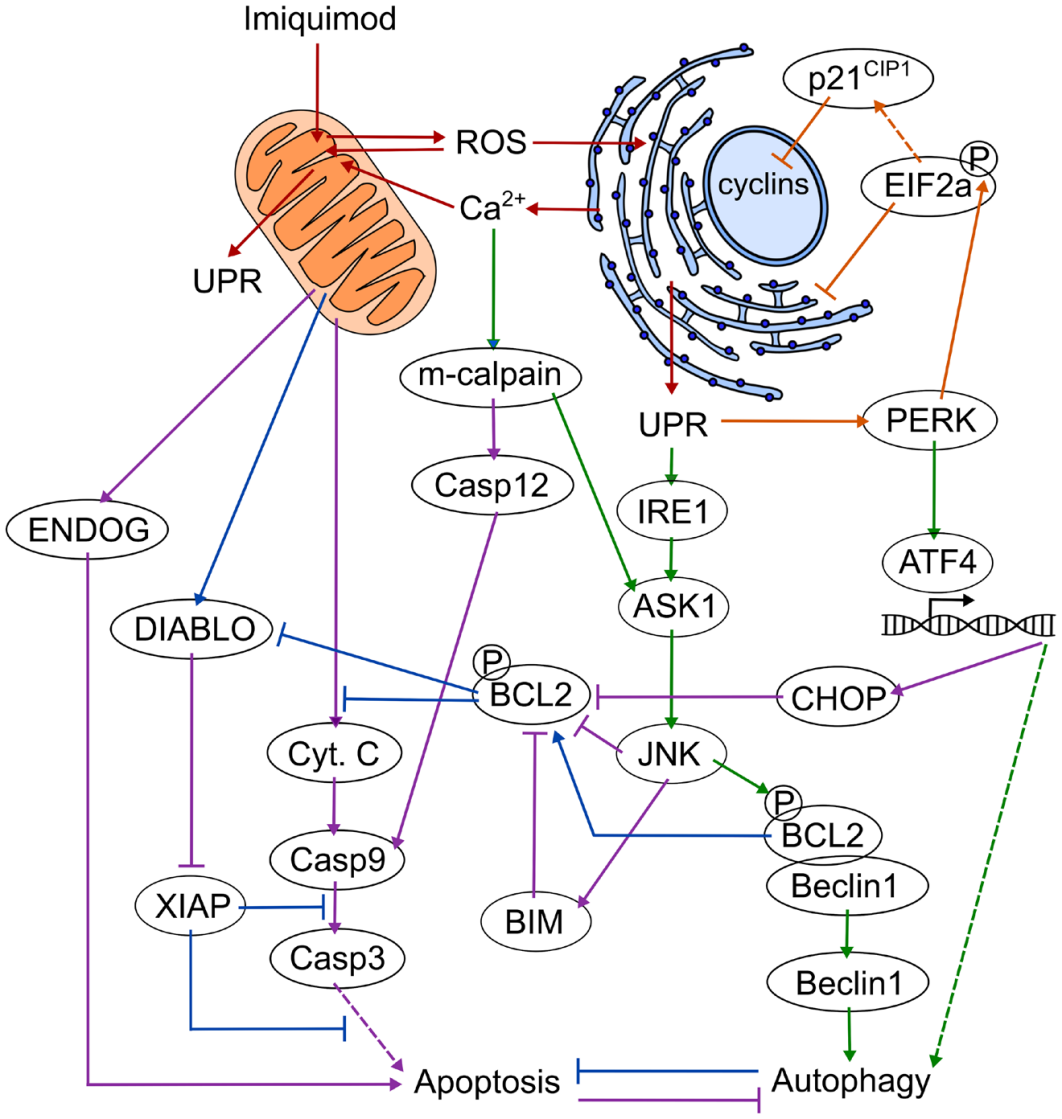
Proposed mechanism of imiquimod action in DFTD cells 1. Induction of cell stress (red arrows): Imiquimod enters the cell and disrupts oxidative phosphorylation at the mitochondria via a TLR7-independent mechanism. ROS is produced and shuttles between the ER and mitochondria to disrupt cellular homeostasis and activate the UPR. Ca^2+^ is depleted from the ER upon activation of ER stress, and is taken up by the mitochondria where it further disrupts oxidative phosphorylation in a stress-induced cycle. 2. Attenuation of protein translation and cell cycle arrest (orange arrows): PERK is activated by the UPR and phosphorylates EIF2a, resulting in attenuation of regular protein translation and suppression of oncogenic pathways. EIF2a induces cell cycle arrest through preferential translation of cyclin inhibitors such as p21^CIP1^. 3. Activation of autophagy (green arrows): PERK stimulates the expression of genes required for stress responses via ATF4, including autophagic genes. IRE1 is activated via the UPR and stimulates JNK via ASK1. Increased cytoplasmic Ca^2+^ activates m-calpain, which further stimulates JNK activity via ASK1. JNK phosphorylates BCL2, resulting in its dissociation from beclin-1, and allowing beclin-1 to induce autophagy. 4. Inhibition of apoptosis (blue arrows): Activation of autophagy inhibits apoptosis, in part through the release of BCL2. BCL2 inhibits the XIAP inhibitor DIABLO and cytochrome C release. DFTD cells survive in this state for up to 72 h. 5. Activation of apoptosis (purple arrows): Prolonged cell stress induces the expression of pro-apoptotic CHOP via the PERK-ATF4 axis. JNK activates pro-apoptotic members of the BCL2 family such as BIM. CHOP, JNK and BIM cooperated to inhibit anti-apoptotic molecules including BCL2. Inhibition of BCL2 activates DIABLO, which inhibits anti-apoptotic XIAP. Cytochrome C is released from the mitochondria, and stimulates the caspase cascade. M-calpain supports the activation of this pathway through cleavage of caspase 12. ENDOG is also released from the mitochondria in response to prolonged oxidative stress and promotes DNA degradation in a caspase-independent manner. DFTD cells undergo apoptosis.

In this study, we have analysed the response of DFT1 cells to imiquimod at the transcriptome and proteome level in order to improve the current understanding of the direct anti-tumor effects of this small molecule in cancer. This analysis has demonstrated that imiquimod disrupts cellular homeostasis, leading to a myriad of molecular changes that are consistent with activation of oxidative and ER stress responses (Figure 7). These changes included positive regulation of PERK, an attenuator of translation at the ER, which likely altered protein production to favour other regulated pro-survival mechanisms such as cell cycle arrest and autophagy. Attenuation of normal protein translation by PERK could have also lead to the negative regulation of tumourigenic pathways, perhaps rendering the cells more susceptible to apoptosis. Lastly, a range of pro- and anti-apoptotic genes involved in ER and oxidative stress responses were also positively regulated in our study, suggesting that the interaction of stress pathways controls the onset of apoptosis in the treated cells. As genes associated with ER stress responses were positively regulated across multiple DFT1 cell lines in our study, and within human cell lines in previous studies, the stress-mediated pathways that were regulated in our study may pose as potential drug targets for enhancement of the direct effects of imiquimod during cancer therapy. This study is the first comprehensive transcriptome and proteome analysis of any imiquimod-treated tumor cell, and is the first proteomic analysis of any cell type of Tasmanian devil origin. Findings from this analysis will allow for improved mechanistic use of imiquimod as a cancer immunotherapy in both human and Tasmanian devil cancer trials.

## SUPPLEMENTARY MATERIALS FIGURES AND TABLES













## Abbreviations

DFTD: devil facial tumor disease
ER: endoplasmic reticulum
TLR: toll-like receptor
pDC: plasmacytoid dendritic cell
ROS: reactive oxygen species
RNA-seq: RNA sequencing
MS: mass spectrometry
GO: gene ontology
UPR: unfolded protein response
IPA: Ingenuity Pathway Analysis
