# Supplementary material for: Transcriptome and proteome profiling reveals stress-induced expression signatures of imiquimod-treated Tasmanian devil facial tumor disease (DFTD) cells
Source: Oncotarget. 2018 Mar 23;9(22):15895–914. doi: 10.18632/oncotarget.24634 (PMC5882306; doi:10.18632/oncotarget.24634)
Supplement: Supplementary file 1 [file oncotarget-09-15895-s001.pdf]

Transcriptome and proteome profiling reveals stress-induced expression signatures of imiquimod-treated Tasmanian devil facial tumor disease (DFTD) cells

SUPPLEMENTARY MATERIALS

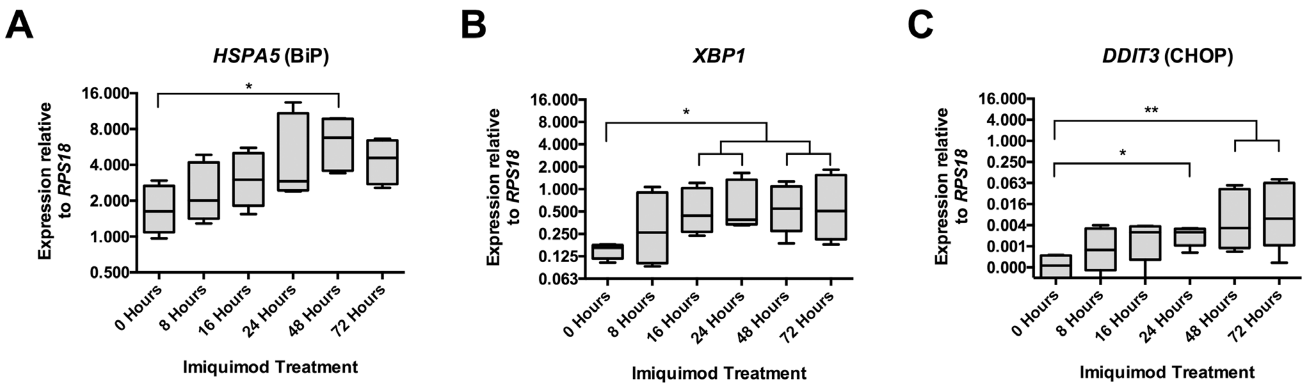

Supplementary Figure 1: Expression of UPR marker genes in DFT1 cell lines over a 72 h period of imiquimod treatment. The DFT1 cell lines C5065, 1426, 4906 and half-pea were treated with imiquimod (60 µg/ml) for 0, 8, 16, 24, 48 or 72 h. Quantitative RT-PCR was performed to measure the expression of the UPR markers *HSPA5*, *XBP1* and *DDIT3* relative to the house-keeping gene *RPS18*. Statistical significance relative to the 0 h sample was measured by one-way ANOVA and Dunnett's multiple comparisons test, and is given by $^{*}p<0.05$, $^{**}p<0.01$ and $^{***}p<0.001$.

Supplementary Table 1: Differential gene expression

See Supplementary File 1

Supplementary Table 2A: GO analysis up-regulated genes
Supplementary Table 2B: GO analysis down-regulated genes

See Supplementary File 2

Supplementary Table 3: Ingenuity canonical pathways

See Supplementary File 3

Supplementary Table 4: Differential expression of proteins

See Supplementary File 4

Supplementary Table 5A: Functional annotation clustering up regulated
Supplementary Table 5B: Functional annotation clustering down regulated

See Supplementary File 5
